# Phenotypic and Genotypic Characterisation of *Burkholderia cenocepacia* J2315 Mutants Affected in Homoserine Lactone and Diffusible Signal Factor-Based Quorum Sensing Systems Suggests Interplay between Both Types of Systems

**DOI:** 10.1371/journal.pone.0055112

**Published:** 2013-01-28

**Authors:** Claudia Udine, Gilles Brackman, Silvia Bazzini, Silvia Buroni, Heleen Van Acker, Maria Rosalia Pasca, Giovanna Riccardi, Tom Coenye

**Affiliations:** 1 Dipartimento di Biologia e Biotecnologie, Università degli Studi di Pavia, Pavia, Italy; 2 Laboratory of Pharmaceutical Microbiology, Ghent University, Ghent, Belgium; Charité-University Medicine Berlin, Germany

## Abstract

Many putative virulence factors of *Burkholderia cenocepacia* are controlled by various quorum sensing (QS) circuits. These QS systems either use *N*-acyl homoserine lactones (AHL) or *cis*-2-dodecenoic acid (“*Burkholderia* diffusible signal factor”, BDSF) as signalling molecules. Previous work suggested that there is little cross-talk between both types of systems. We constructed mutants in *B. cenocepacia* strain J2315, in which genes encoding CepI (BCAM1870), CciI (BCAM0239a) and the BDSF synthase (BCAM0581) were inactivated, and also constructed double *(*Δ*cepI*ΔBCAM0581, Δ*cciI*ΔBCAM0581 and Δ*cepI*Δ*cciI*) mutants and a triple (Δ*cepI*Δ*cciI*ΔBCAM0581) mutant. Subsequently we investigated phenotypic properties (antibiotic susceptibility, biofilm formation, production of AHL and BDSF, protease activity and virulence in *Caenorhabditis elegans*) and measured gene expression in these mutants, and this in the presence and absence of added BDSF, AHL or both. The triple mutant was significantly more affected in biofilm formation, antimicrobial susceptibility, virulence in *C. elegans*, and protease production than either the single or double mutants. The ΔBCAM0581 mutant and the Δ*cepI*ΔBCAM0581 and Δ*cciI*ΔBCAM0581 double mutants produced significantly less AHL compared to the WT strain and the Δc*epI* and Δc*ciI* single mutant, respectively. The expression of *cepI* and *cciI* in ΔBCAM0581, was approximately 3-fold and 7-fold (p<0.05) lower than in the WT, respectively. The observed differences in AHL production, expression of *cepI* and *cciI* and QS-controlled phenotypes in the ΔBCAM0581 mutant could (at least partially) be restored by addition of BDSF. Our data suggest that, in *B. cenocepacia* J2315, AHL and BDSF-based QS systems co-regulate the same set of genes, regulate different sets of genes that are involved in the same phenotypes and/or that the BDSF system controls the AHL-based QS system. As the expression of the gene encoding the C6-HSL synthase CciI (and to a lesser extent the C8-HSL synthase CepI) is partially controlled by BDSF, it seems likely that the BDSF QS systems controls AHL production through this system.

## Introduction

*Burkholderia cenocepacia* is a member of the *Burkholderia cepacia* complex (Bcc), a group of at least 17 closely related species [1]. Bcc species are opportunistic pathogens causing severe infections in immunocompromised patients, and in people with chronic granulomatous disease or cystic fibrosis (CF) [2]. Infection with *Burkholderia cenocepacia* can lead to the development of “cepacia syndrome” and is associated with increased morbidity and mortality [3]. The *B. cenocepacia* epidemic ET12 lineage that originated in Canada and spread to Europe in the 1980s has been one of the most prevalent Bcc genotypes isolated from CF patients, with strain J2315 being studied as model isolate [4].

The production of many factors thought to be important for virulence in this organism is controlled by quorum sensing (QS), a cell-cell signalling mechanism that allows bacteria to communicate with neighbouring cells, in order to coordinate gene expression [5]. In Gram-negative bacteria *N*-acyl homoserine lactone (AHL) molecules are the most commonly used signals and they are produced by a LuxI-type synthase. A LuxR transcriptional regulator binds the cognate molecule at high threshold levels, altering the gene expression [6]. *B. cenocepacia* has two of these QS systems: CepIR, which is present in all Bcc species, and CciIR, which has been found only in *B. cenocepacia* containing the cenocepacia island (cci) (i.e. in ET12 strains) [5], [7]–[10]. Both systems appear to be maintained during chronic infections in CF patients [11]. CepI primarily synthetizes oxanoyl-homoserine lactone (C8-HSL) and minor amounts of hexanoyl-HSL (C6-HSL), whereas CciI is primarily responsible for the synthesis of C6-HSL with lesser amounts of C8-HSL [8]. The CepIR and CciIR systems regulate expression of a large number of genes involved in traits important for virulence, such as motility, biofilm formation, secretion, extracellular enzymes and antimicrobial drug resistance [5], [12]–[14]. These two QS systems are organised in a hierarchical way, as CepR regulates the expression of the *cciIR* operon [8], [10].

Recently, another signal molecule, *cis*-2-dodecenoic acid (“*Burkholderia* diffusible signal factor”, BDSF), has been identified in *B. cenocepacia* [15]. BDSF is a structural analogue of *cis*-11-methyl-2-dodecenoic acid, a QS signal known as diffusible signal factor (DSF) in the plant bacterial pathogen *Xanthomonas campestris* [16]. Synthesis of *cis*-2-dodecenoic acid requires the presence of BCAM0581, which encodes an enzyme related to RpfF, the DSF synthase of *X. campestris* [15]–[17]. BDSF is synthesized from a fatty acid synthetic intermediate, the acyl carrier protein (ACP) thioester of 3-hydroxydodecanoic acid. This intermediate is intercepted by the RpfF homolog, a bifunctional protein that not only catalyses the dehydration of 3-hydroxydodecanoyl-ACP to *cis*-2-dodecenoyl-ACP, but also cleaves the thioester bound, resulting in the free acid [18]. Although much remains to be learned about the BDSF signal transduction mechanism, the sensor kinase BCAM0227 appears to be involved in the signal perception [19]. A *B. cenocepacia* J2315 mutant in which BCAM0581 was inactivated showed reduced *in vitro* biofilm formation, as well as reduced virulence in the *Galleria mellonella* infection model [20]. Reduced expression of genes encoding putative virulence factors as well as reduced mortality in a zebrafish model were also reported for this mutant [21]. BDSF inhibits *Candida albicans* hyphal growth *in vitro*, suggesting a potential role of this molecule in competition between *B. cenocepacia* and *C. albicans in vivo* [15]. In addition, BDSF produced by *B. cenocepacia* can influence biofilm formation and polymyxin resistance in *P. aeruginosa* [22], [23].

At the moment little is known about the interplay between the various QS systems in *B. cenocepacia*. Deng *et al*. reported that expression levels of *cepI* and *cciI* were not affected in the BCAM0581 deletion mutant and showed that the addition of synthetic BDSF to a *cepR* mutant restored the expression of several virulence genes to wild type (WT) levels [21]. These data suggest that both types of QS systems regulate a similar set of genes in parallel. Also in 2009, Ryan *et al*. reported that the signalling function of BDSF in *B. cenocepacia* is independent of AHL-based signalling as mutation of BCAM0581 did not alter the levels of AHL molecules being produced [20]. In addition, Ryan *et al.* reported that mutations in *cepI* or *cepR* did not give rise to the same phenotypic alterations as mutating BCAM0581, although detailed information was not presented. A comparison of the BDSF regulon with the genes regulated by the *cepIR* or *cciIR* systems identified only minimal overlap [19]. In addition, the expression of neither BCAM0227 nor BCAM0581 appeared to be affected in *B. cenocepacia* K56-2 Δ*cepR*, Δ*cciR* and Δ*cepR*Δ*cciIR* mutants [5]. This suggests that there is little cross-talk between both types of systems, although firm evidence is still missing.

In the present study we have explored this issue further. To this end we constructed mutants in which genes encoding CepI (BCAM1870), CciI (BCAM0239a) and the BDSF synthase (BCAM0581) were inactivated, and also constructed double *(*Δ*cepI*ΔBCAM0581, (Δc*ciI*ΔBCAM0581 and Δ*cepI*Δ*cciI)* and a triple (Δ*cepI*Δ*cciI*ΔBCAM0581) mutant, and investigated phenotypic properties as well as gene expression in these mutants.

## Results and Discussion

### Deletion of Genes BCAM1870, BCAM0239a and BCAM0581

In order to investigate the interplay between the different QS systems in *B. cenocepacia*, deletion mutants were constructed for *cepI*, *cciI* and BCAM0581 (alone or in combination), following the protocol described by Hamad *et al.* [24]. *B. cenocepacia* J2315 is difficult to manipulate genetically, because of its intrinsic antibiotic resistance, which prevents the use of the most common selectable markers for gene exchange [25]. The mutagenesis strategy used in the present study makes use of the expression of the I-*SceI* endonuclease, thus allowing the creation of markerless deletions and inactivation of more than one gene in the same strain. To start constructing the inactivated strains, the *cepI* gene was deleted in *B. cenocepacia* J2315, obtaining the single mutant Δc*epI*. Subsequently, the Δ*cepI* mutant was cured from the plasmid pDAI-*SceI*-SacB by growing the strain in LB medium without antibiotics and then screening the resulting colonies for loss of tetracycline resistance. Subsequently, a new mutation could be introduced in the same strain. Using the same strategy, BCAM0581 gene was deleted in Δ*cepI*, leading to a double mutant (Δ*cepI*ΔBCAM0581). Subsequently, the double mutant was cured and it was possible to inactivate a third gene, and a triple mutant (Δ*cepI*Δ*cciI*ΔBCAM0581) was obtained. In addition, we constructed two other single-gene mutants starting from WT *B. cenocepacia* J2315, defective in *cciI* and in BCAM0581, respectively. Finally, the cured Δ*cepI* and ΔBCAM0581 mutants were independently used to delete the *cciI* gene, obtaining two different double mutants: Δ*cepI*Δ*cciI* and Δ*cciI*ΔBCAM0581, respectively. The presence of the correct deletions in each strain was confirmed by PCR analysis and sequencing (data not shown).

### Biofilm Formation

To confirm that any differences observed between the different strains was not due to pronounced differences in growth rate, we determined growth curves for all strains in the various media used in the present study. No significant differences in growth curves could be observed in MH (data not shown).

**Figure 1:**
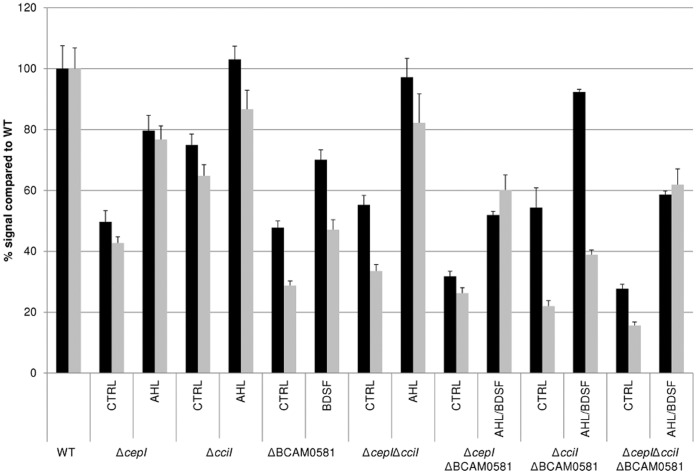
Relative amount of biofilm formation in the various strains compared to WT (average±standard deviation). Biofilm were formed for 24 h in the absence (CTRL) and presence of signalling molecules (AHL, BDSF or both; 5 µM). N = 60 for all experiments. Black bars: relative amount of metabolically active cells as quantified with CellTiter Blue. Grey bars: relative amount of total biomass as quantified with crystal violet.

In order to determine whether there were quantitative differences in biofilm formation, biofilms of the different strains were grown in 96-well microtiter-plates and biofilms were quantified using crystal violet staining as well as a resazurin-based viability staining. For the single mutants our data (Fig. 1) confirm previous observations, i.e. all single mutants form considerably less biofilm than the WT strain (p<0.001). However, we also noted that this reduction in biofilm formation was more pronounced for the double ΔcepIΔBCAM0581 and the triple ΔcepIΔcciΔBCAM0581 mutants than for the single mutants (p<0.001). To confirm the role of BDSF and/or AHL in the observed biofilm phenotype, we repeated the experiment with the QS-mutants but added BDSF (5 µM) and/or AHL (5 µM) to the growth medium. Our data confirm that addition of signalling molecules restored the biofilm phenotype (Fig. 1).

**Figure 2:**
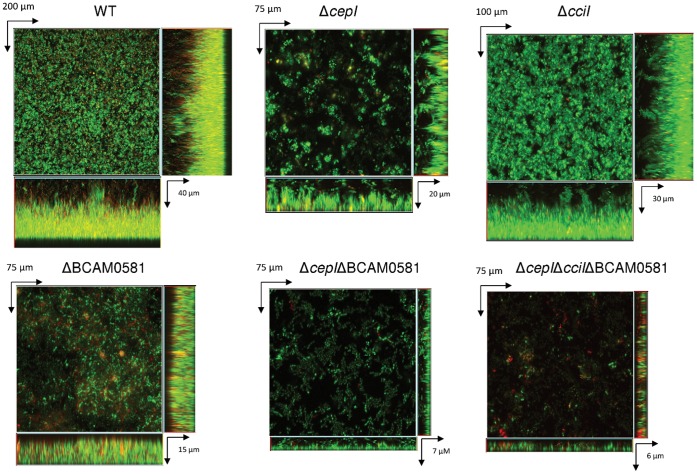
Representative confocal images of 24-h-old biofilms formed by various *B. cenocepacia* QS mutants.

The differences in biofilm formation between the different strains were also obvious from CLSM experiments (Fig. 2). While the WT strain formed a thick, densely-packed biofilm, biofilms formed by any of the single mutants were much thinner and less-densely packed. The double and triple mutants formed very thin biofilms that covered only a small part of the surface.

### Antimicrobial Susceptibility

To assess the susceptibility of planktonic cells of the WT and mutant strains to ciprofloxacin, meropenem, tobramycin, ceftazidime and cefoxitine, MICs were determined according to EUCAST guidelines (Table 1). While the Δ*cepI*, Δ*cciI* and Δ*cepI*Δ*cciI* mutants did not show much changes in the susceptibility profile compared to the WT, the ΔBCAM0581 mutant was considerably more sensitive to ceftazidime and meropenem. This was also the case for the Δ*cepI*ΔBCAM0581 double and Δ*cepI*Δ*cciI*ΔBCAM0581 triple mutant, which in addition were also considerably more sensitive to cefoxitine than the WT strain. The ΔBCAM0581 mutant, as well as the double and triple mutants also showed a slightly increased susceptibility towards tobramycin (2-fold decrease in MIC).

**Table 1:** MIC (µg/ml) of various antibiotics towards *B. cenocepacia* J2315 WT and mutant strains.

MIC (µg/ml) for					
Strain	CAZ	FOX	CIP	MEM	TOB
J2315	>128	>128	8	32	256
Δc*epI*	>128	>128	8	32	256
Δ*cepI*+AHL	>128	>128	8	32	256
Δ*cciI*	64	128	8	32	256
Δ*cciI*+AHL	128	>128	8	32	256
ΔBCAM0581	32	128	8	8	128
ΔBCAM0581+ BDSF	64	>128	8	16	256
Δ*cepI*ΔcciI	>128	>128	8	32	128
Δ*cepI*ΔcciI +AHL	>128	>128	8	32	256
Δ*cepI*ΔBCAM0581	32	64	8	8	128
Δ*cepI*ΔBCAM0581+ AHL/BDSF	64	128	8	16	256
Δ*cciI*ΔBCAM0581	32	128	8	16	128
Δ*cciI*ΔBCAM0581+ AHL/BDSF	64	>128	8	32	256
Δ*cepI*Δ*cciI*ΔBCAM0581	16	64	8	8	128
Δ*cepI*Δ*cciI*ΔBCAM0581+ AHL/BDSF	32	128	8	16	256

CAZ, ceftazidime; FOX, cefoxitin; CIP, ciprofloxacin; MEM, meropenem; TOB, tobramycin.

**Figure 3:**
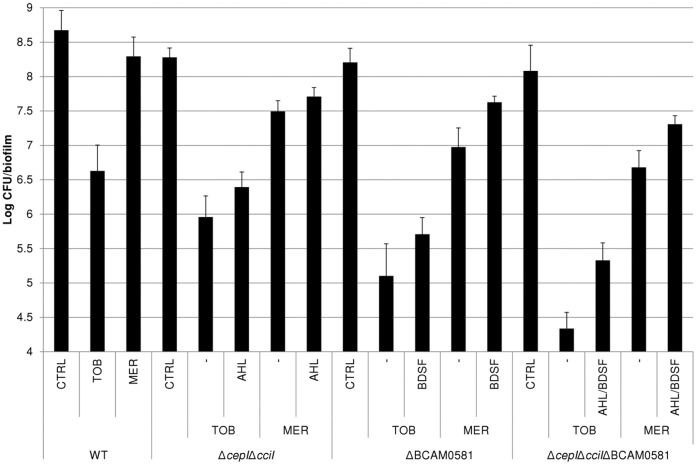
Effect of treatment of mature biofilms with tobramycin (A) or meropenem (B). Antibiotics were added in a concentration equal to 4×MIC for each strain to 24 old biofilms, grown in the absence (CTRL) or presence of signalling molecules (AHL, BDSF or both; 5 µM). Data are expressed as average log (CFU/biofilm) (±standard deviation). N ≥3 for all experiments. Treatment of the mutants resulted in significantly higher reductions than treatment of the WT (p<0.05). In addition, supplementation resulted in a significant decrease in susceptibility compared to the unsupplemented mutant (p<0.05).

In addition, we assessed the antimicrobial susceptibility of sessile cells, by treating mature biofilms with tobramycin or meropenem. In order to treat biofilms formed by the various strains with an equivalent concentration of the antibiotic, we decided to treat them with a concentration that equals four times the MIC for each strain. The results are shown in Fig. 3. Treatment of WT biofilms with tobramycin resulted in a reduction in cell numbers of 2.05±0.38 log units, which is in agreement with previously published data [26]. Tobramycin had slightly higher effect on biofilms formed by the Δ*cepI* and Δ*cciI* single mutants (data not shown). However, reductions were most pronounced for the mutants defective in BDSF and/or AHL signal production (reductions of 3.57±0.24, 2.71±0.31 and 4.34±0.47 log units for ΔBCAM0581, Δ*cepI*Δ*cciI* and, Δ*cepI*Δ*cciI*ΔBCAM0581 respectively) (Fig. 3). Reductions obtained for the WT biofilm treated with meropenem were much lower than those observed with tobramycin, again in agreement with previously published data [26]. However, also for meropenem the reductions were more pronounced for QS mutants (Fig. 3). In addition, supplementing the QS-mutant strains with BDSF and/or AHL resulted in a significant (p<0.05) decreased susceptibility towards both antibiotics in comparison to the unsupplemented mutant strain (Fig. 3, Table 1).

### Effect of Mutations in QS Systems on Survival of Infected *C. elegans*

**Figure 4:**
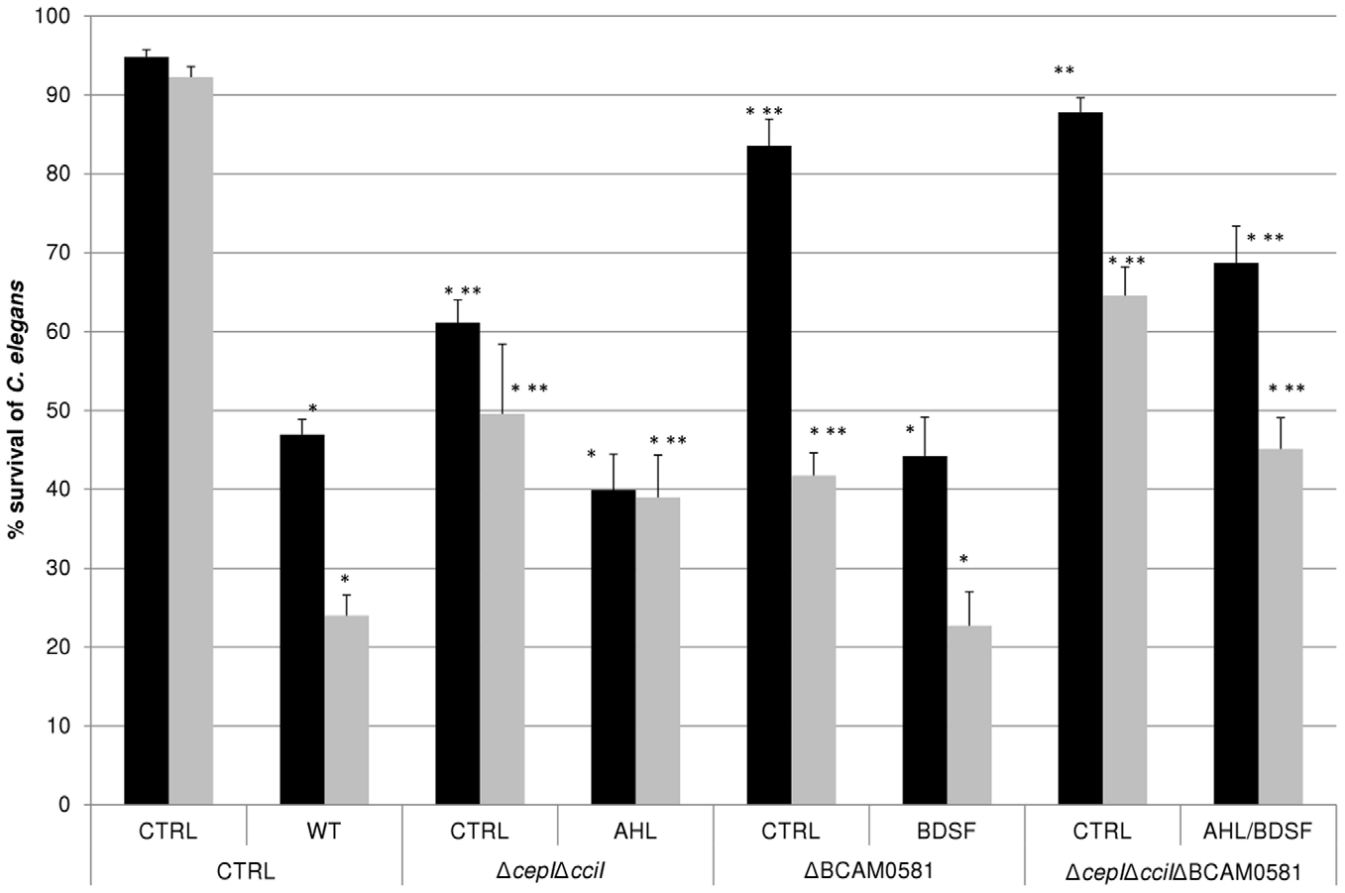
Percent survival of *C. elegans* (average ± standard deviation) infected with various *B. cenocepacia* strains in the absence (CTRL) or presence of signalling molecules (AHL, BDSF or both; 5 µM). The results are expressed as the percent survival after 24 h (black bars) or 48 h (grey bars) of infection and treatment. *: significantly different survival compared to uninfected control (p<0.0001); **: significantly different survival compared to infection with WT (p<0.0001).

In agreement with previous observations that a *B. cenocepacia* ΔBCAM0581 mutant was less virulent in the *G. mellonella* [20] and zebrafish [21] infection models, we observed that survival in *C. elegans* infected with this mutant, the Δ*cepI*Δ*cciI* double mutant or the Δ*cepI*Δ*cciI*ΔBCAM0581 triple mutant was significantly higher than survival of WT-infected nematodes (Fig. 4). After 24 h of infection, survival in WT infected worms dropped to 47±13%, while survival of the worms infected with the ΔBCAM0581 (84±11% survival), the Δ*cepI*Δ*cciI* (61±12% survival), or triple mutant (88±12% survival) were significantly higher. In addition, survival after 24 h was not significantly different between uninfected *C. elegans* and *C. elegans* infected with the triple mutant. After 48 h, only 24±19% of the worms survived the infection with WT, while survival in populations infected with the ΔBCAM0581, Δ*cepI*Δ*cciI* or triple mutant was significantly higher (42±15%, 50±26% and 65±25% survival, respectively). While significantly higher than the survival observed in populations infected with the WT strain (p<0.0001), it is noteworthy that this is still lower than the survival in the uninfected control (92±8%). In addition, survival rates after 24 h were not significantly different between the WT strain and the strains defective in AHL or BDSF signalling when these strains were supplemented with the respective signalling molecules, which indicates that supplementation resulted in a total restoration of virulence towards *C. elegans* (Fig. 4). Finally, although still significantly different from the WT, supplementation of the QS mutants with BDSF, AHL or both resulted in a significant decrease in survival after 48 h compared to the unsupplemented mutant strains (Fig. 4).

### Production of AHL Signalling Molecules is Regulated by BDSF

**Figure 5:**
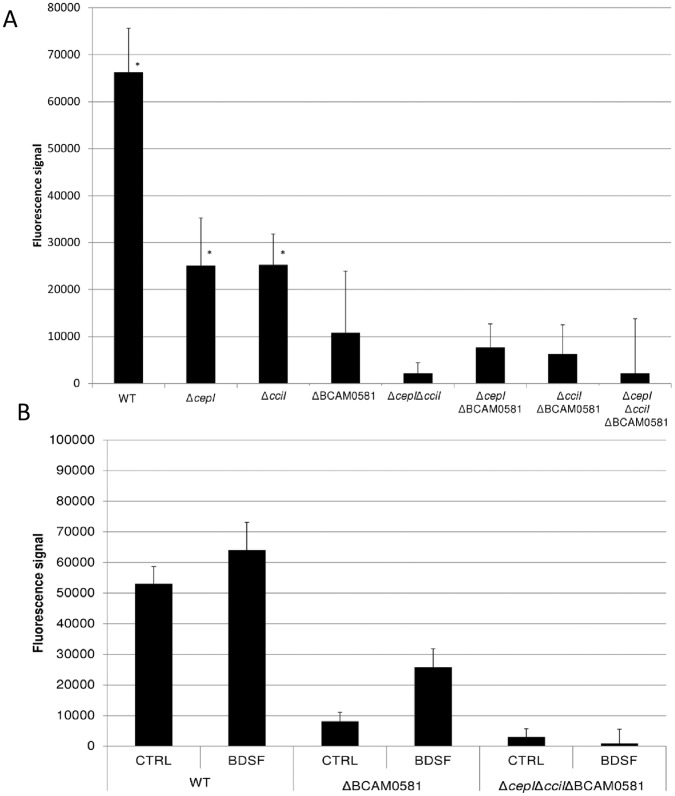
Production of AHL molecules by various *B. cenocepacia* strains A) in the absence and B) presence of BDSF (5 µM). *: significantly different from no AHL production (p<0.001); **: significantly different from control receiving no BDSF (p<0.001).

**Figure 6:**
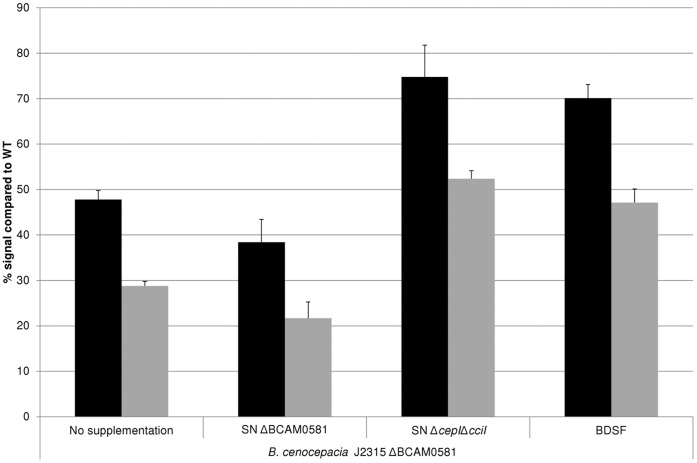
Production of BDSF by the Δ*cepI*Δ*cciI* double mutant. Biofilm formation of the ΔBCAM0581 without supplementation, supplemented with supernatant of the Δ*cepI*Δ*cciI* mutant or ΔBCAM0581 mutant or supplemented with BDSF (5 µM). The relative amount of metabolically active cells was quantified with CellTiter Blue (black bars), while the relative amount of total biomass was quantified with crystal violet (grey bars).

**Figure 7:**
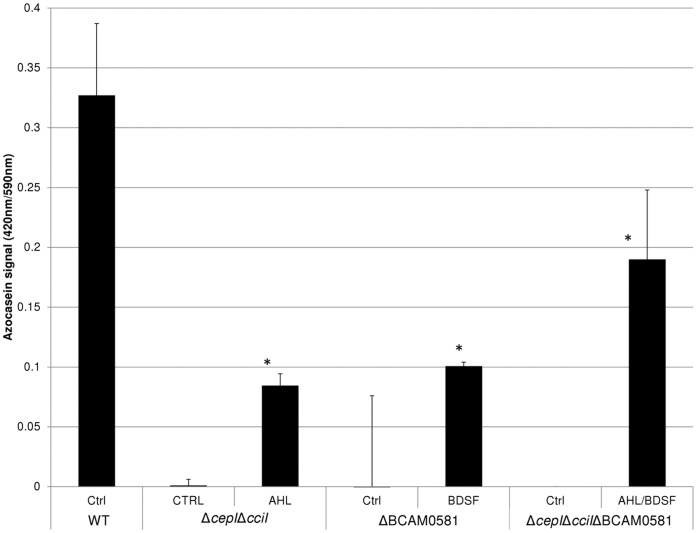
Protease production in various *B. cenocepacia* strains in the abscence (CTRL) and presence of AHL (5 µM), BDSF (5 µM) or both AHL/BDSF (5 µM). *: significantly different protease production compared to no signal (p<0.05).

We then wanted to determine whether BDSF signalling influences the production of AHL QS molecules. To this end the production of AHL was measured using the *E. coli* JB523 biosensor, and this for various *B. cenocepacia* strains grown in the presence or absence of BDSF (Fig. 5). Supernatant of the mutants nor WT supernatant displayed toxicity towards the *E. coli* biosensor. As such we can exclude that differences are observed due to differences in growth of the biosensor. In agreement with our expectations, the WT strain produced copious amounts of AHL, while none were being produced by the triple mutant (which lacks both AHL synthases, as well as BDSF synthase). As expected, addition of BDSF (5 µM) did not significantly alter the production of AHL in these strains. However, to our surprise, we noticed that the ΔBCAM0581 strain produced significantly less AHL than the WT (p<0.001). Addition of BDSF (5 µM) to the growth medium of this mutant partially restored the production of AHL (p<0.001 for comparison between ΔBCAM0581 with and without BDSF). In contrast, supernatant of the Δ*cepI*Δ*cciI* mutant could restore biofilm formation when added to the ΔBCAM0581 mutant (Fig. 6). This not only indicates that BDSF is involved in co-regulating AHL-controlled phenotypes but also that this effect seems to be uni-directional. To confirm this we subsequently investigated whether addition of signalling molecules could restore protease production in the various mutants. Protease production was previously shown to be controlled by AHL-based signalling [12], [27]. Protease production was significantly (p<0.05) lower in the QS mutants compared to the WT, and the addition of AHL (5 µM), BDSF (5 µM) or both resulted in an increased protease production in the Δ*cepI*Δ*cciI*, ΔBCAM0581 and Δ*cepI*Δ*cciI*ΔBCAM0581 mutant, respectively (Fig. 7). To further elucidate the role of BCAM0581 and BDSF in the production of AHL at the genomic level, we measured the expression of *cepI* (BCAM1870), *cepR* (BCAM1868), *cciI* (BCAM0239a), *cciR* (BCAM0240), *zmpA* (BCAS0409), *lipA* (BCAM0949), *lipB* (BCAM0950) and *orbI* (BCAL1696) by qPCR. Gene expression was measured in planktonic cells in the presence or absence of added signalling molecules. Although no difference in *cepR* gene expression was observed (Table 2), the expression of *cepI, cciI* and *cciR* was significantly downregulated in the ΔBCAM0581 and Δ*cepI*Δ*cciI*ΔBCAM0581 mutant compared to the WT (p<0.05). Moreover, addition of BDSF to the ΔBCAM0581 mutant or AHL and BDSF to the Δ*cepI*Δ*cciI*ΔBCAM0581 mutant resulted in an increased expression of *cepI, cciI* and *cciR*. In addition, *zmpA*, *lipA* and *orbI* were significantly downregulated in the ΔBCAM0581, Δ*cepI*Δ*cciI* and Δ*cepI*Δ*cciI*ΔBCAM0581 mutants, while *lipB* was only downregulated in the Δ*cepI*Δ*cciI* and Δ*cepI*Δ*cciI*ΔBCAM0581 mutant. Supplementation with the corresponding signal resulted in a significant increase in the transcription of *zmpA*, *lipA*, *lipB* and *orbI* in the QS mutants. Our results are different from those previously observed [20] : Ryan et al. showed that BDSF affects the expression of virulence genes in *B. cenocepacia* J2315, but claimed that this effect is independent of AHL signalling because the BCAM0581 deletion did not affect AHL levels. However, it was not immediately clear which approach was used to detect AHL levels, and as such it is difficult to speculate on what caused this difference. Deng et al. [21] previously reported no difference in expression of *cepI* or *cepR* in their BCAM0581 mutant. However, they did not measure AHL levels, nor did they measure the expression of the second AHL synthase *cciI*.

**Table 2:** Expression of the QS-genes *cepI/R* and *cciI/R* and virulence genes *zmpA, lipA*, *lipB* and *orbI* in the ΔBCAM0581, Δ*cepI*Δ*cciI* and triple mutant in the absence (CTRL) and presence of signal molecules (5 µM).

Gene name or ID	Fold changes compared to *B. cenocepacia* J2315 WT					
	Δ*BCAM0581*		Δ*cepI*Δ*cciI*		Δ*cepI*Δ*cciI*Δ*BCAM0581*	
	CTRL	+BDSF	CTRL	+AHL	CTRL	+BDSF/AHL
*cepI*	−3.3*	1.1¶	ND	ND	ND	ND
*cepR*	−2.0	−1.6	−1.1	−1.2	1.7	−1.9¶
*cciI*	−7.2*	−1.9¶	ND	ND	ND	ND
*cciR*	−2.5*	−1.5¶	−1.4	1.8	−2.6*	−1.4¶
*zmpA*	−18.1*	−2.2* ¶	−12.6*	−1.6¶	−17.9*	−1.2¶
*lipA*	−9.6*	−1.9¶	−3.1*	−1.6¶	−3.4*	1.3¶
*lipB*	−2.0	2.2¶	−8.8*	−4.3* ¶	−4.2*	−1.1¶
*orbI*	−2.2*	2.6* ¶	−2.1	1.0¶	−4.3*	3.0* ¶

ND: not detected.

* significantly different gene expression compared to WT (p<0.05).

¶ significantly different gene expression compared to gene expression in the absence of supplementation with AHL, BDSF or AHL/BDSF (p<0.05).

Recently Schmid *et al*. [28] described that the AHL and BDSF QS systems in *B. cenocepacia* strain H111 (a strain lacking the *cciIR* system) operate in parallel to control specific as well as overlapping sets of genes. A first working model proposed by Schmid *et al*. assumes an unknown c-di-GMP receptor protein that stimulates transcription of target genes. An alternative working model assumes the two QS cascades converge and regulate a yet unknown common regulator, which is in turn responsible for the regulation of the expression of target genes. Their data are largely in accordance with results from the present study that indicate that the BDSF QS system co-regulates the transcription of several virulence genes as well as AHL production in *B. cenocepacia* J2315. However, whether the BDSF system is directly regulating the AHL system in *B. cenocepacia* J2315 or whether a unknown regulator or cascade mechanism is involved to link both systems is currently unknown.

### Conclusions

Our data show an “additive” effect of a mutation in the BDSF QS system of *B. cenocepacia* on several AHL-controlled phenotypes, with the triple mutant being more affected in biofilm formation, antimicrobial susceptibility, virulence in *C. elegans*, and protease production than either the single or double mutants. This suggests that both types of QS systems co-regulate the same set of genes, regulate different sets of genes that are involved in the same phenotypes and/or that one type of QS system regulates the other. The BDSF synthase mutant produces less AHL molecules than the WT, but AHL production can partially be restored by adding BDSF, suggesting that the BDSF QS system is involved in regulating AHL-based QS. This was confirmed by our observation that the expression of the gene encoding the C6-HSL synthase CciI (and to a lesser extent the gene encoding the C8-HSL synthase CepI) is partially controlled by BDSF.

## Materials and Methods

### Strains and Growth Conditions

Bacterial strains and plasmids used in this work are listed in Table 3. Bacteria were grown in Luria-Bertani (LB) broth (Difco, Basingstoke, UK), with shaking at 200 rpm, on LB agar, or in Mueller-Hinton (MH) broth (Oxoid, Detroit, MI) at 37°C. *Escherichia coli* OP50 was grown in TSB (Oxoid) at 37°C, whereas *E. coli* JB523 was cultured at 30°C in LB medium containing 4% NaCl. *Caenorhabditis elegans* N2 (*glp-4; sek-1*) was propagated under standard conditions, synchronized by hypochlorite bleaching, and cultured on nematode growth medium using *E. coli* OP50 as a food source, as described previously [29], [30].

**Table 3:** Strains and plasmids used.

Strain/plasmid	Properties	Source/reference
***B. cenocepacia*** ** strains**		
J2315	WT	[4]
Δ*cepI*	J2315 ΔBCAM1870	This study
Δ*cciI*	J2315 ΔBCAM0239a	This study
ΔBCAM0581	J2315 ΔBCAM0581	This study
Δ*cepI*Δ*cciI*	J2315 ΔBCAM1870ΔBCAM0239a	This study
Δ*cepI*Δ*BCAM0581*	J2315 ΔBCAM1870ΔBCAM0581	This study
Δ*cciI*ΔBCAM0581	J2315 ΔBCAM0239aΔBCAM0581	This study
Δ*cepI*Δ*cciI*ΔBCAM0581	J2315 ΔBCAM0239aΔBCAM1870ΔBCAM0581	This study
***E. coli*** ** strains**		
DH5a	F^−^Φ80d*lacZ*Δ*M15*Δ (*lacZYA-argF*)*U169 endA1 recA1 hsdR*17(r_K_^−^ m_K_^+^) *supE44 thi*-*1* Δ*gyrA96 relA1*	Laboratory stock
SY327	*araD* Δ (*lac pro*) *argE*(Am) *recA56 nalA* λ *pir*, Rif^r^	M. Valvano
OP50	WT	Laboratory stock
JB523	pJBA130 Tet^r^ pME6031-*luxR-P_luxI_*-RBSII-*gfp*mut3^/−^T_0_–T_1_	[35]
**Plasmids**		
pGEM-T Easy	Vector for PCR cloning, Amp^r^	Promega
pGPI*Sce*-I-Xcm	*ori*_R6K_, ΩTp^r^, *mob* ^+^, containing the I*Sce*-I restriction site, Tp^r^, Cat^r^, *xylE*	M. Valvano
pRK2013	*ori*_colE1_, RK2 derivative, Kan^r^, *mob* ^+^, *tra* ^+^, Kan^r^	M. Valvano
pDAI*Sce*-I-*sacB*	pDA12 encoding the I*Sce*-I homingendonuclease, Tet^r^, *sacB*	M. Valvano

Amp^r^, ampicillin resistance; Cat^r^, chloramphenicol resistance; Kan^r^, kanamycin resistance; Rif^r^, rifampin resistance; Tet^r^, tetracycline resistance; Tp^r^, trimethoprim resistance.

### Mutagenesis

The mutagenesis procedure was performed as described by Hamad et al. [24]. Briefly, the upstream and downstream DNA sequences (about 500 bp each) that flank the gene targeted for deletion were cloned into pGPISce-I-Xcm. This suicide plasmid contains a unique restriction site for the endonuclease I-*SceI* and the *xylE* reporter gene. The PCR amplification of flanking regions for the construction of the mutagenesis plasmids were performed with the HotStar HiFidelity Polymerase kit (Qiagen, Milan, Italy), and the specific amplification conditions were optimized for each primer pair (Table 4). For the deletion of BCAM1870, we used primer pairs KOcepIXL-KOcepIBL and KOcepIBR-KOcepIKR. For the deletion of BCAM0239a, PCR amplification of the flanking regions was performed using primer pairs KOcciIXL-KOcciIBL and KOcciIBR-KOcciIKR. For the deletion of BCAM0581, primer pairs KO0581XL-KO0581BL and KO0581BR-KO0581KR were used. Mutagenesis plasmids were mobilized by conjugation into *B. cenocepacia* J2315 WT**,** Δ*cepI,* ΔBCAM0581 and Δ*cepI*ΔBCAM0581 strains. Exconjugants were selected in the presence of trimethoprim (200 µg/ml), chloramphenicol (400 µg/ml) and ampicillin (200 µg/ml). To distinguish true cointegrants from colonies that spontaneously became resistant to chloramphenicol and/or trimethoprim, plates were sprayed with catechol since in the presence of this compound colonies expressing 2,3-catechol-dioxygenase encoded by *xylE* turn bright yellow [24]. Subsequently, a second plasmid, pDAI-*SceI*-SacB (encoding the I-*SceI* endonuclease) was introduced by conjugation. Site specific double-strand breaks take place in the chromosome at the I-*SceI* recognition site, resulting in tetracycline-resistant (due to the presence of pDAI-*SceI*-SacB) and trimethoprim-susceptible (indicating the loss of the integrated mutagenic plasmid) exconjugants. In this case we used tetracycline (250 µg/ml) and ampicillin (200 µg/ml) for selection of exconjugants. The desired gene deletions were confirmed by PCR amplification and sequencing, using primer pairs COcepIFL-COcepIRR, COcciIFL-COcciIRR and CO0581FL-CO0581RR for BCAM1870, BCAM0239a and BCAM0581, respectively (Table 4).

**Table 4:** Primers used in this work.

Primer name	Primer sequence	Restriction enzyme^a^
KOcepIXL	5′-TTCTAGAGGCTATACCGAATGGC-3′	*Xba*I
KOcepIBL	5′-TTGGATCCGCGAGTTCGTGTGGCA-3′	*Bam*HI
KOcepIBR	5′-TTGGATCCAGCAGGTAGATGGGCG-3′	*Bam*HI
KOcepIKR	5′-TTGGTACCCGATGGGAACGGGCAG -3′	*Kpn*I
KOcciIXL	5′-TTTCTAGATTGCCTTATACGACCA-3′	*Xba*I
KOcciIBL	5′-TTGGATCCATCGTCCAGCCTACCT-3′	*Bam*HI
KOcciIBR	5′-TTGGATCCGTAACTGCCAACCCAG-3′	*Bam*HI
KOcciIKR	5′-TTGGTACCCGCTTGAACTCTCCCC-3′	*Kpn*I
KO0581XL	5′-TTTCTAGAACCTTCATCACGGCGA-3′	*Xba*I
KO0581BL	5′-TTGGATCCATGGACATCACGGAAG-3′	*Bam*HI
KO0581BR	5′-TTGGATCCTCAGTTGCGAGAGTTC-3′	*Bam*HI
KO0581KR	5′-TTGGTACCGCGTGTGGTCCTGTTC-3′	*Kpn*I
COcepIFL	5′-TTGTCAGGTTTCAGTA-3′	
COcepIRR	5′-TGGCAGGGCAGGCGGA-3′	
COcciIFL	5′-ATTCTTCCGTCAGCCA-3′	
COcciIRR	5′-TCTCGCCAGTCCGTCG-3′	
CO0581FL	5′-CGAAGGGGCTCGGCAT-3′	
CO0581RR	5′-CCAGCGGGAAGGAGAT-3	
CepI FW (qPCR)	5′-TCCCGTCGGCGAACGAAAGT-3′	
CepI RV (qPCR)	5′-CGGCGAACAGCGACTTCAGC-3′	
CepR FW (qPCR)	5′-ACGGCTGGATGGCGCACTAC-3′	
CepR RV (qPCR)	5′-ACGCCCACCGACAATCCGAA-3′	
CciI FW (qPCR)	5′-TCGACGACATGCCAACCACGA-3′	
CciI RV (qPCR)	5′-CCGCTCCGGGTAACTGCCAA-3	
CciR FW (qPCR)	5′-GCTGGCCACCGCCTTTCTCA-3	
CciR RV (qPCR)	5′-AGCGGACACGTCACCGAACA-3′	
ZmpA FW (qPCR)	5′-GCGGCGGCTCGGTCTAC-3′	
ZmpA RV (qPCR)	5′-CGGGATCGTTCGGGTTGTTCG-3′	
LipA FW (qPCR)	5′-AACCGCGCCCGCCGACGACTAT-3′	
LipA RV (qPCR)	5′-GCCCTGGCTGTGACCGACGAGATT-3′	
LipB FW (qPCR)	5′-GCCGGCGTCGCGATGTGGAG-3′	
LipB RV (qPCR)	5′-GCGCGGTCAGGCAATAGTCG-3′	
OrbI FW (qPCR)	5′-ACGCGTGCATTGCTGGGTCTGTTC-3′	
OrbI RV (qPCR)	5′-GCGCGGCCGTCGTATGCT-3′	
BCAM2784FW (qPCR)	5′-CCCCGTTCTCGCTCTACGT-3′	
BCAM2784 RV (qPCR)	5′-GTGTCGCCGAGGCAGAAAT-3′	
BCAS0175 FW (qPCR)	5′-ATGGCCAGTTCGCTCATCA-3′	
BCAS0175 RV (qPCR)	5′-ACGCGATGTCGATACTCGAAT-3′	
BCAL2694 FW (qPCR)	5′-CTTGCCGTGATCCTCGAGAT-3′	
BCAL2694 RV (qPCR)	5′-GAGATCAGCGAGGCCGAGTA-3′	

a Restriction endonuclease sites incorporated in the oligonucleotide sequences are underlined.

### Determination of Minimal Inhibitory Concentrations (MIC)

MICs of antibiotics were determined in triplicate according to the EUCAST broth microdilution protocol, using flat-bottom 96-well microtiter plates (TPP, Trasadingen, Switzerland) [26]. The range of antibiotic concentrations was from 0.25 to 128 mg/l for ciprofloxacin, meropenem, ceftazidime and cefoxitine; for tobramycin higher concentrations were tested (between 2 and 1024 mg/l). The inoculum was standardized to approximately 5×10^5^ CFU/ml. The plates were incubated at 37°C for 20 h, and the optical density at 590 nm was determined by using a multilabel microtiter plate reader (Envision; Perkin-Elmer LAS, Waltham, MA). The lowest concentration of antibiotic for which a similar optical density was observed in the inoculated wells and the blank control wells was recorded as the MIC.

### Biofilm Formation in 96-well Microtiterplates

Strains were grown overnight in MH until OD_590 nm_ = 0.2 (approx. 10^8^ CFU/ml). 100 µl of the bacterial suspension were transferred to the wells of a round bottomed 96-well microtiter plate (TPP). Bacteria were allowed to adhere and grow without agitation for 4 h at 37°C. After 4 h, plates were emptied and washed with sterile physiological saline (PS). After this washing step, all wells were filled with 100 µL of MH and the plate was incubated for 20 h at 37°C. The number of metabolically active (i.e. living) cells in the biofilm was determined with a resazurin-based assay [31]. In brief, wells were rinsed after 24 h biofilm formation and 100 µl PS was added, followed by addition of 120 µl CellTiter-Blue (CTB) solution (Promega, Leiden, The Netherlands). After 1 h at 37°C, fluorescence (excitation and emission filters of 486 nm and 535 nm, respectively) was measured using an Envision plate reader. The total biofilm biomass was quantified by crystal violet (CV) staining, as described previously [31]. In brief, plates were rinsed with sterile PS, biofilms were fixed by adding 100 ml 99% methanol for 15 min, after which the methanol was removed and plates were air-dried. Biofilms were then stained with 100 µl of 0.1% CV (Pro-Lab Diagnostics, Richmond Hill, ON, Canada). After 20 min, CV was removed, and wells were filled with 150 ml 33% acetic acid (Sigma Aldrich, Bornem, Belgium). After 15 min the absorbance was measured at 590 nm using an Envision plate reader.

### Biofilm Formation on Silicone Disks

For quantification of the numbers of sessile cells, *B. cenocepacia* strains were allowed to form biofilms on silicone disks (Q7-4735; Dow Corning, Midland, MN) in a 24-well plate as previously described [32]. In brief, 1 ml of bacterial suspension was transferred to the wells of a 24-well plate (TPP) containing a sterile silicone disk. Bacteria were allowed to adhere and grow without agitation for 4 h at 37°C. After 4 h, growth medium was removed and the plates were washed with PS. Subsequently, all wells were filled with 1 ml of MH and the plates were incubated for 20 h at 37°C. After 24 h, biofilms were rinsed once with PS and subsequently treated with an antibiotic or a blank control solution. After 24 h at 37°C, the biofilms were rinsed with PS. Sessile cells were removed from the disks by three cycles of vortexing (30 s) and sonication (30 s; Branson 3510; Branson Ultrasonics Corp., Danbury, CT), and the number of CFU/biofilm was determined by plating the resulting suspensions.

### Confocal Laser Scanning Microscopy (CLSM)

For CLSM analysis, 100 µl of 10^8^ CFU/ml bacterial suspension was transferred to the wells of a black flat-bottom 96-well plates with glass bottom. Negative controls received 100 µl of MH. The plates were incubated at 37°C for 4 h and the medium was removed. The wells were washed with 100 µl of PS and filled with 100 µl of MH. Subsequently the plates were incubated at 37°C for 20 h. Afterwards, the wells were rinsed with PS and filled with 100 µl of a SYTO9 solution (containing 1 ml of PS and 3 µl of SYTO9). The plates were incubated for 15 min at room temperature and the biofilm was visualized with a Nikon C1 confocal laser scanning module attached to a motorized Nikon TE2000-E inverted microscope (Nikon Benelux, Brussels, Belgium) equipped with a Plan Apo VC60×1.4 NA oil immersion objective and suitable optical elements to obtain fluorescent and differential interference contrast (DIC) transmission images. SYTO9 was excited with the Argon ion 488 nm laser line and emission light was collected using a 500–530 nm band pass filter and Z-stacks were recorded. Tests were performed on at least two wells for each situation and representative images are shown.

### Quantification of AHL and Protease Production

AHL production was quantified using the *E. coli* JB523 assay in a 96-well microtiterplate as previously described [33]. In brief, an overnight culture of the reporter strain was diluted in fresh sterile LB medium containing 4% (w/v) NaCl to an OD_590 nm_ = 0.1 and 100 µl of this cell suspension was added to the wells of a black 96-well microtiterplate (Perkin Elmer). Tetracycline was added to a final concentration of 100 µg/ml. Activation of QS by AHL was tested by addition of C6-HSL (20 µM) (Sigma). Sterile MilliQ water served as a negative control. To study the production of AHL signals, 100 µl of supernatant of a bacterial suspension were added. The microtiterplate was then incubated for 24 h at 30°C and fluorescence from GFP expression was measured at λ_ex_ 475 nm and λ_em_ 515 nm, using a Victor Wallac^2^ multilabel counter (Perkin Elmer). The protease activity assay was conducted as previously described [34].

### *C. elegans* Survival Assay

The *C. elegans* survival assay was carried out as described previously [32]. In brief, synchronized worms (L4 stage) were suspended in a medium containing 95% M9 buffer, 5% brain heart infusion broth (Oxoid), and 10 µg of cholesterol (Sigma-Aldrich) per ml. 0.5 ml of this suspension of nematodes was transferred to the wells of a 24-well microtiter plate. An overnight bacterial culture was centrifuged, resuspended in the assay medium, and standardized to 10^8^ CFU/ml. Next, 250 µl aliquots of this standardized suspension were added to each well and the assay plates were incubated at 25°C for up to 2 days. The fraction of dead worms was determined by counting the number of dead worms and the total number of worms in each well, using a dissecting microscope.

### Measuring Gene Expression by Real-time Quantitative PCR

Planktonic cells were grown as described above in the absence and presence of BDSF (5 µM) for 24 h. Treated and untreated cells were harvested and transferred to sterile tubes. RNA was extracted immediately following harvesting of the cells, using the Ambion RiboPure Bacteria Kit (Ambion, Austin, TX) according to the manufacturer’s instructions and included a DNAse I treatment for 1 h at 37°C. cDNA was synthesized using the QScript cDNA Supermix (Quanta, Gaithersburg, MD). Forward and reverse primers were developed using tools available on the NCBI website and were compared with the *B. cenocepacia* J2315 genome sequence using BLAST to determine their specificity (Table 3). The primer concentration used was 300 nM. All qPCR experiments were performed using the PerfeCTa SYBR Green Fastmix (Quanta) on a Bio-Rad CFX96 Real-Time System C1000 Thermal Cycler. Each sample was spotted in duplicate and control samples without cDNA were included in each experiment. The initial 30 s denaturation step at 95°C was followed by 40 amplification cycles, consisting of 5 s at 95°C and 30 s at 60°C. A melting curve analysis was included at the end of each run. To allow accurate normalization of our data, we also included three reference genes (BCAL2694, BCAS0175, BCAM2784) for which the stable expression was confirmed in preliminary experiments (data not shown).
